# Lived Experiences of Occupational Therapy Students From Under‐Represented Backgrounds in Professional Educational Programs: A Scoping Review

**DOI:** 10.1155/oti/8173486

**Published:** 2026-08-18

**Authors:** Alesia Ford, Diane L. Smith, Tamara Wanklyn, Danielle Hitch

**Affiliations:** ^1^ Children′s Friend and Services Early Intervention, Providence, Rhode Island, USA; ^2^ MGH Institute of Health Professions, Boston, Massachusetts, USA, mghihp.edu; ^3^ Deakin University, Melbourne, Australia, deakin.edu.au; ^4^ Western Health, Footscray, Australia, westernhealth.org.au

**Keywords:** diversity, health professional education, higher education, equity, inclusion, intersectionality

## Abstract

**Objective:**

The aim of this scoping review is to describe the experiences of occupational therapy students from diverse backgrounds during professional education. The objective is to identify common facilitators, barriers, or strategies that impact their safety and inclusion in professional education and ultimately the workforce and to highlight any gaps in the literature.

**Introduction:**

Occupational therapy lacks diversity in its workforce, which is predominantly White and female in many countries. Increased workforce diversity is a priority for the occupational therapy profession to enhance the provision of culturally competent care and address health inequity and access barriers. Therefore, recruiting and retaining occupational therapy students from diverse backgrounds in a safe and inclusive academic environment is important for the entire profession.

**Methods:**

A scoping review of sources exploring the lived experiences of occupational therapy students from one or more diverse backgrounds was undertaken. The sources used qualitative or mixed methods approaches and were published in English since 2014. Databases searched via the EBSCOHost platform included Medline, CINAHL, AMED, Pubmed, SocINDEX, ERIC, and CENTRAL via Cochrane. Citation searching was conducted on included papers using the Connected Papers platform. Articles were screened by two independent researchers by title and abstract, and then by full text, before relevant data were extracted. The initial title search identified 1494 articles from databases and 85 additional sources from citation searching. After applying the inclusion criteria and removing duplicates, 1020 articles were subjected to further screening, and 165 studies were selected for full‐text review.

**Results:**

The final number of studies included in the scoping review was 24. Thematic analysis identified evidence relating to barriers and facilitators in the systemic and organizational context, departmental and peer supports, and the students′ lived experiences of professional programs. Strategies suggested to address these barriers include purposeful mentoring, increased knowledge of the student′s culture, and increased accessibility.

**Conclusion:**

There has been an increase in the amount of research published regarding diversifying student populations. The experiences of diverse occupational therapy students during professional education are influenced by multiple individual, group, organizational, and systemic factors. More research is needed to evaluate the effectiveness of these strategies.

## 1. Introduction

The demographics of the occupational therapy workforce in many countries often fail to reflect the population they serve [1]. These communities are diverse and intersectional, encompassing individuals of different genders, sexes, ages, socioeconomic statuses, cultural backgrounds, religions, sexual orientations, neurodiversity, and living locations, as well as people with and without disabilities [2]. In contrast, occupational therapists typically identify as White, middle‐class, and female [1, 3, 4]. In this paper, the terms “diverse” and “diversity” are used to represent the wide variety of different human characteristics within human populations, such as race, ethnicity, gender, sexual orientation, age, socioeconomic status, religion, physical and mental abilities, and cultural background. Groups which differ from majority characteristics (in this case, White, middle‐class, and female) are often characterized “under‐represented,” “marginalized,” and “vulnerable.” [5]

Limited diversity within healthcare professional workforces can lead to a lack of cultural humility when interacting with diverse communities [5]. A lack of diversity in the workforce may reduce incentives and opportunities for people from these groups to join the profession, particularly when the visibility of individuals from these groups is low, and few role models exist [6]. This is not only an equity issue, but it may also be influencing the demographic composition of student cohorts entering higher education to study occupational therapy [6]. Increasing opportunities for students from diverse groups to enter and remain in the profession may enhance recruitment and retention [7].

The experiences of people from diverse groups can be both positively and negatively influenced by the safety and inclusivity of the educational environment and support available [6]. Safe environments include those perceived as welcoming and enabling of positive and inclusive interactions that promote social and emotional learning [8]. Some research has been conducted to explore the educational environment in occupational therapy courses around the world. Previous research into the experiences of diverse occupational therapy students is generally single case studies of specific groups, for example, occupational therapists in Canada who were trained overseas [9], allied health students moving to study in a regional university [10], and new graduate occupational therapists commencing work in rural Australia [11].

A recent scoping review exploring the intersectional experiences of minoritized occupational therapy practitioners and students found that practitioners and students alike often tolerate hostile and unfair environments, recommending that the profession focus on and improve efforts to address these issues [12]. The authors synthesized information about the articles, such as the country, group investigated, and whether the authors identified as members of these groups, and developed four themes from the literature [12]. However, the experiences of students and practitioners were evaluated together, though the needs of these two groups may differ when addressing any challenges identified and warrant separate evaluation [12]. The review also did not synthesize literature regarding barriers, facilitators, and implications for the higher education sector and did not include studies about other minoritized groups, such as students from rural backgrounds or those from low socioeconomic backgrounds [12]. This present article contributes to the literature by providing results specifically related to occupational therapy students. This article will contribute to the literature by a more intentional and expansive synthesis of barriers and facilitators related to the student experience, as well as suggestions from students and these authors. Exploring occupational therapy students′ experiences in educational settings reported in the literature will provide a profile of their level of inclusion in higher education contexts and will allow for the examination of what strategies should be implemented to improve the support available to these students.

Occupational therapy students may belong to multiple diverse groups; therefore, this review was conducted using an intersectional lens to examine issues experienced by students. Intersectionality is a framework that can be used to understand how different factors or identities overlap, compound, or intersect to create a profile of discrimination and privilege that may influence someone′s life experiences [13]. Intersectionality is a relatively recent concept in the occupational therapy literature and has most often been applied to service users rather than students or clinicians [14]. However, adopting an intersectional perspective is crucial to developing a more nuanced and person‐centered understanding of the needs of diverse occupational therapy students and the implications for the broader profession. This article provides a valuable contribution to the occupational therapy literature by using an intersectional approach to analyze the data and to develop strategies to address issues identified by the review and the voices of the students.

A recent scoping review of experiences of diversity for occupational therapy practitioners and students found that both groups are exposed to and must tolerate hostile and unfair educational and employment environments, leading the authors to recommend that the profession prioritize efforts to address these issues [12]. The aim of this scoping review was to step beyond the current literature and analyze current knowledge gaps and identify and synthesize potential strategies for increasing diversity in the occupational therapy student population, specifically. The primary objective was to identify common facilitators, barriers, or strategies that impact safety and inclusion in the workforce, using an intersectional approach.

Therefore, the research question for this review is “What does the literature say about the lived and living experiences of occupational therapists from diverse backgrounds during professional education using an intersectional lens?” Subquestions include the following: (1) What are the common facilitators and barriers to safety and inclusion to support diverse occupational therapists through professional education? (2) What strategies are suggested to support the safety and inclusion of occupational therapists through professional education? and (3) From an intersectional perspective, how do the experiences of occupational therapy students from under‐represented and diverse backgrounds compare?

## 2. Methods

The review was conducted per the Joanna Briggs Institute (JBI) methodology for scoping reviews [15]. A scoping review was the most appropriate approach due to the relatively sparse literature available on the topic and the need to understand future directions for education, research, practice, and policy [16]. The scope of the review was intentionally broad, incorporating data on the experiences of people from various and/or intersecting diverse groups to examine the experiences of occupational therapy students from multiple perspectives. The Preferred Reporting Items for Systematic reviews and Meta‐Analyses extension for Scoping Reviews (PRISMA‐ScR) was used to structure all reporting [16]. The review was registered in the Open Science Framework registry on 14/11/2024 (10.17605/OSF.IO/3CWYF) [17].

This review was part of a broader review that included diverse occupational therapists at all stages of their professional career (i.e., from professional education to practice). During the review process, it became evident that the context and experiences of students were distinct enough from those of practitioners, and the sources related to these two groups were analyzed separately. The findings of sources, including both students and practitioners, were included in both reviews, reporting only those relevant to the chosen group. The scoping review related to diverse occupational therapists in practice is published separately.

### 2.1. Inclusion Criteria

#### 2.1.1. Participants

Sources appropriate for this scoping review included articles that focused on occupational therapy students from diverse backgrounds from any area of practice or geographical location. All sources addressed the concept of the lived and living experiences of occupational therapy students, including the exploration of perceptions, attitudes, beliefs, subjective data, narratives, or any other approach to understanding personal experience. Studies were also included if they contained a mixed sample of practitioners and students, and if specific findings related to student participants were reported.

#### 2.1.2. Types of Sources

Included sources reported on the experiences of students from any population group characterized as diverse, marginalized, under‐represented, or vulnerable. These groups included those who identify as disabled, male, nonbinary, LGBTIQ+, culturally and linguistically diverse, living rurally or remotely, Indigenous or First Nations people, people of color, adults over 65 years, and/or of lower socioeconomic status.

Only qualitative studies were included. This included all types of qualitative studies, including, but not limited to, phenomenological, grounded theory, ethnographic, descriptive, action research, and feminist research designs. Mixed methods studies were also included, where qualitative approaches were the primary methodology.

### 2.2. Search Strategy

A search strategy involved locating published studies relevant to the review question, which began on November 14, 2024, and was periodically updated until the final review on June 30, 2025. Studies published in English since 2014 were included to encompass developments in multiple healthcare systems internationally and university occupational therapy programs in the last decade. The first step was to complete a limited search of MEDLINE (via the EBSCOhost platform) to identify key articles on the topic. (see protocol for details [17]). In the second step, keywords contained in the titles and abstracts of relevant articles, along with index terms, provided the basis for developing a full search strategy for MEDLINE (EBSCO) (see Supporting Information II). The third step involved citation searching of all included sources of evidence, which was conducted using the Connected Papers platform [18]. A university librarian was repeatedly consulted to review and provide feedback on the search strategy to ensure that its scope was feasible and comprehensive [19]. The scope was broadened for any stage of a professional career and eventually split into an article relevant to students and another relevant to practitioners.

### 2.3. Evidence Screening and Selection

**Figure 1:**
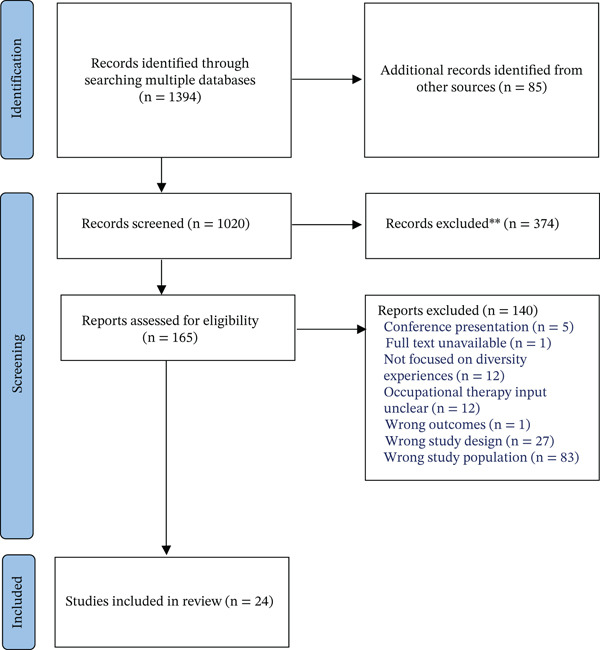
PRISMA‐ScR diagram of record retrieval. Source. Figure format from Tricco, A. C., Lillie, E., Zarin, W., O′Brien, K. K., Colquhoun, H., Levac, D., … Straus, S. E. (2018). PRISMA Extension for Scoping Reviews (PRISMA‐ScR): Checklist and Explanation. Annals of Internal Medicine, 169, 467–473. 10.7326/M18-0850

Following the search, all identified citations were uploaded into the Covidence platform [20], and duplicates were automatically removed. Titles and abstracts were screened by two independent reviewers within the team (A.F., D.S., and T.W.) for assessment against the inclusion criteria for the review [20]. Two independent reviewers then assessed the full text in detail against the inclusion criteria. Reasons for the exclusion of sources at this stage were recorded and are reported here (see Figure 1). Disagreements between reviewers at each stage of the selection process, typically less than 10% of sources, were resolved through discussion with a third reviewer. The search and screening results are presented in a PRISMA flow diagram (see Figure 1) [15].

### 2.4. Data Charting Process

Data were extracted from included sources by all authors independently (A.F., D.S., T.W., and D.H.) using the data extraction tool developed by the reviewer (see Supporting Information III). The data extraction table was developed based on examples from other reviews and recommendations [21]. A draft data extraction form was piloted with all review team members independently comparing their results to ensure that the form captured relevant data for the review question. Extracted data included details about the participants, concept, context, study location, study methods, and key findings relevant to the review questions.

### 2.5. Data Analysis and Presentation

Critical appraisal of the articles was not conducted, consistent with scoping review methodology. Study characteristics (e.g., population and publication year) were described numerically and narratively. All data were organized, prepared, and read through multiple times to generate codes. Codes were developed by listing topics and clustering similar topics and analyzed as expected codes, surprising codes, and codes of unusual or conceptual interest. These codes were used to organize the data and were turned into categories, which were then developed into themes. Inter‐researcher consensus about themes was addressed through independent development of themes and then discussion to determine consensus [21]. Descriptive thematic analysis was then conducted across studies, synthesizing literature related to the research questions. These data were reported narratively, with supporting tables and charts. Themes were developed through analysis of recurring issues that emerged in the literature that were identified and agreed upon by the authors [21].

## 3. Results

The initial titles search identified 1394 articles from databases and 85 additional sources from citation searching. After applying the inclusion criteria and removing duplicates, 1020 articles were subjected to further screening, and 165 studies were selected for full‐text review (see Figure 1).

**Figure 2:**
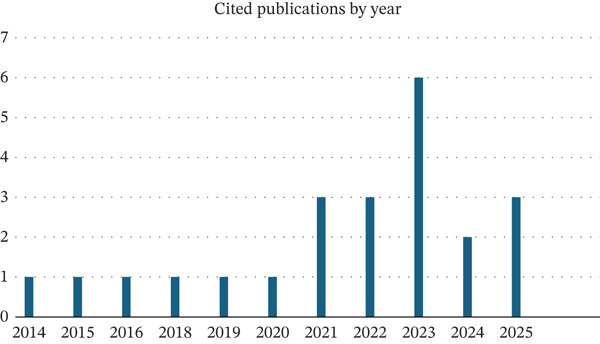
Cited publications by year of publication.

The final number of studies included in this scoping review was 24. Eighteen articles focused on the experiences of professional education of diverse occupational therapy students, with six further including both occupational therapy students and practitioners. The range of the sample was 1–200 with a mean of 39.37, a median of 13, and a mode of 7. The most frequently examined student groups were students of color (*n* = 8), international students (*n* = 6), and students with a disability (*n* = 5). The most common study designs included phenomenological (*n* = 11), structured interviews (*n* = 3), and scoping reviews (*n* = 2). Figure 2 illustrates the number of publications per year, showing a general upward trend. Of the 24 publications, the majority of the studies originated in Canada (*n* = 9, 37.5%), followed by the United States (*n* = 7, 29.2%), Australia (*n* = 3, 12.5%), United Kingdom (*n* = 3, 12.5%), and South Africa (*n* = 2, 8.3%).

### 3.1. Themes

Thematic analysis of the sources identified evidence related to barriers and facilitators in the systemic and organizational context; department and peer supports are presented in Table 1. From this review, a summary of the lived experiences with thematic references bolded in each study is presented in Table 2.

**Table 1:** Study characteristics, barriers, and facilitators.

Author/year, country, journal	Aim	Methodology/data collection	Participants	Barriers to safety, inclusion, and/or justice	Facilitators, strategies, or resources for safety, inclusion, and/or justice	Limitations
Aldridge et al. (2023). Canada *Journal of Occupational Therapy Education, 7* (4).	To explore Black OT students′ lived experiences with systemic racism during their academic program	Phenomenological research design	15 Black OT students attending school in the United States.	Students identified increased anxiety around the lack of Black representation and reported feeling burdened for having to initiate change as the “diversity person.”	Participants initiated change by starting chapters for the Coalition of Occupational Therapy Advocates for Diversity (COTAD), opening forums and workshops to spark conversation surrounding diversity, and working with professors to develop more culturally sensitive materials.	Interviews were conducted via Zoom due to safety concerns regarding the global COVID‐19 pandemic. These interviews may have felt impersonal and may have skewed body language, facial expressions, and the natural flow of conversation. Connectivity issues may also have affected these interviews. The methodology is somewhat flawed as the data are self‐reported and filtered through the participant′s point of view. The final limitation includes researcher bias.
		15 one‐to‐one semistructured interviews and five focus groups were conducted.				
Benitez et al. (2022). United States *Journal of Occupational Therapy Education*	To identify and understand the perspectives of international students and alumni on their transition to graduate‐level occupational therapy education and daily life in the United States.	Qualitative research design	13 international students and alumni currently enrolled in postprofessional occupational therapy programs in the United States.	Identified barriers included changes in every aspect of daily life, and that each experience is new, different, and extraordinarily complex, affecting their overall well‐being.	Creation of a support system: a home away from home. They also wanted to give back to the home country: improving occupational therapy education, practice, and research.	The results presented were from a single university and may not accurately represent the views or experiences of all international students. This study includes perspectives of international students from the Philippines, Ireland, India, South Korea, Singapore, Venezuela, Canada, China, South Africa, and Taiwan, and lacks perspectives of students from other parts of the world.
		11 individual interviews and one joint interview with two individuals were conducted.				
		Data were analyzed through group‐based methods.				
Bevan, J. (2014) United Kingdom *Disability & Society*	To describe a small‐scale study of the experiences of disabled occupational therapists, recounting their journey and having a first‐hand account of the reality of being a disabled health professional.	Ethnographic research design.	1 = diagnosed with Parkinson′s disease 1 = born with cerebral palsy 1 = dyslexia. 1 = hearing impairment and rheumatoid arthritis. 1 = (the researcher) childhood epilepsy, which reoccurred as an adult.	Environmental barriers included steps or stairs, absence of hearing loops or accessible information. The participants did not see environments as a barrier; the environment was seen as a barrier by others, Organizational barriers indicated a general lack of knowledge relating to funding sources for equipment, adaptations, and support.	A firm commitment to removing disabling barriers in education, practice, and research, and recognizing and endorsing initiatives like the Disability Forum and reviewing their guidelines on an ongoing basis. A positive, inclusive approach towards disabled occupational therapists may inspire greater diversity within the profession, challenging its stereotypical image to meet the needs of current and future registrants	This study only examined the retrospective experiences of a small number of members of the Disability Forum, and the findings are therefore not generalizable to all disabled occupational therapists, but may be transferrable to some, as well as to other disabled health professionals or students. The study suggested that barriers still exist to the acceptance of disabled people as professional colleagues.
		Individual, unstructured, ethnographic interviews were used.				
		A thematic analysis was employed, involving the reading and annotation of transcripts to identify themes.				
Ching, E. & Ammon. A. (2020). United States *Diversity & Equality in Health and Care*	This research study explored the lived experiences of a recent graduate of color and a faculty of color in a mentoring relationship.	Phenomenological research design	Two participants: one student of color who recently graduated and one faculty of color.	This study identified a lack of representation as a barrier to success and inclusion in academic settings.	The findings of this study focus on the need for quality mentorship, spaces, and opportunities to dialogue about race and racism, and institutional structural support for the recruitment, retention, and success of both students and faculty of color in academia	Small sample size
		Semistructured interviews were used for this study.				
Easterbrook, et al. (2015). Canada *Disability & Society*	To explore the experiences of students in Health and Human Service programs with disabilities.	Phenomenological research	12 students with disabilities in HHS programs: (medicine, *n* = 1; nursing, *n* = 2; occupational therapy, *n* = 1; physical therapy, *n* = 1; social work, *n* = 2; teacher education, *n* = 5)	Students found there were often complexities around the disclosure of disability.	Faculty can foster a learning environment that is supportive of students′ differing levels of comfort concerning telling others about their disabilities and needed accommodations.	Small exploratory study in which students may not be representative of all students with visible and nonvisible disabilities.
		Semistructured and structured interviews				
Edelist et al. (2024). Canada *Medical Education*	To examine how the Health and Human Services education creates meanings of disability, accommodations, and professional competence, to investigate how students with disabilities continue to experience barriers.	Phenomenological research design	35 HHS students with disabilities	The dominant medical model of disability in Health and Human Service education creates institutional barriers that require students to constantly (re)construct their “professional” identity in relation to their “patient” identity.	Suggestions for strategies included reworking competency standards to critically examine taken‐for‐granted competency and licensing standards, particularly the assumption that disability is a problem for practice. Strategies suggested training to shift ableist ideas about what it means to be a competent practitioner.	The study′s structure resulted in some HHS professions being disproportionately represented in the data, and the view may not be reflective of students with disabilities from other professions.
		14 individual and seven group interviews were conducted.				
		Interpretive analysis				
Ford, et al. (2021). United States *American Journal of Occupational Therapy*	To explore the perceived challenges to and facilitators of the recruitment and retention of OTPs and students of color.	Interpretive, constructionist design	Five occupational therapy practitioners and students of color in the United States.	Participants reported feelings of self‐doubt and tokenization within the education setting. Students identified nonwelcoming settings, racist faculty, and systematic barriers such as financial support as barriers to success.	Purposive recruitment targeting diverse institutions, along with holistic admission and diversity‐driven missions. Creation of formal mentoring programs via diversity caucuses. Cultural awareness/bias training for faculty/clinical instructors; inclusive curricula highlighting diverse OTPs.	This study had a small sample size, and its findings may not be representative of the views of all practitioners and students of color. Participant recruitment was limited through social media, word of mouth, and at the in‐person conference.
		Data were collected online from three focus groups and four interviews.				
Gross, E. et al. (2023). Canada *International Journal of Inclusive Education*	To understand the barriers experienced by students with disabilities in Canadian education during practice placement, and the means that might facilitate support and accommodations, from the perspectives of students with disabilities.	Mixed methods, quantitative and qualitative design	82 survey participants from health and human services and occupational therapy programs in Canada. Of the 82, 11 were interviewed.	Students identified a lack of knowledge and awareness of supports regarding placement accommodations. Students were unaware of their university′s procedures for seeking placement accommodations. Students expressed concern about academic educators′ lack of awareness of the existence of, or the options for, placement accommodations.	Collaboration across Health and Human Service programs to develop clear and effective placement accommodation procedures. Those who support disabled students in placement should receive education on supporting students with disabilities and legal obligations of accommodation.	Study limitations included a small qualitative sample size.
		Semistructured interviews.				
Hendricks, F. & Toth‐Cohen, S. (2018). South Africa *Occupational Therapy International*	To examine the personal narratives of Black OT students attending the Occupational Therapy International National Student Leadership Camp, designed to foster leadership development.	Pilot study. Structured interviews	12 occupational therapy students from six university OT programs in South Africa	Within the historical context of a contested description of being “Born Frees” (i.e., born into Democratic South Africa), students face significant challenges from the macro environment in terms of socioeconomic‐political struggles, requiring positive psychological capital.	Use of personal storytelling in OT leadership camps/curricula, to facilitate leadership growth in self‐knowledge, self‐awareness, and other elements of mindful leadership. Leadership development models that recognize life purpose, values, and morality.	This small pilot study may not be representative of the experiences of Black student leaders in OT.
Jarus, T. et al. (2023). Canada *Medical Education*	To examine the recurrent forms of social relations that underlie the participation of individuals with disabilities across health professions within the current context of health education and practice.	Phenomenological design, utilizing grounded theory	56 individuals with disabilities: health practitioners (*n* = 29) and students (*n* = 27) from five professions: medicine (*n* = 6), nursing (*n* = 15), occupational therapy (*n* = 13), physical therapy (*n* = 7), and social work (*n* = 15).	Challenges to legitimacy and the physical inaccessibility of many practice and education settings alienate students with disabilities. Educational settings that are not accessible put the responsibility on disabled students to do the extra planning to engage in learning activities.	Participants described supportive relationships with colleagues, employers, instructors, and others in health education and practice as respectful, collaborative, trusting, kind and warm, rigorous, but fair and welcoming, sensitive to needs, open, encouraging, and friendly.	Study participants came from only two Canadian provinces and may not capture experiences common in other jurisdictions
		Semistructured interviews,				
Lalor, A. F. et al. (2019). Australia *British Journal of Occupational Therapy*	To understand international students′ perceptions of the purpose of practice placements and the attributes that contribute to successful practice education.	Qualitative design	Seven international students from countries located in the Asia‐Pacific region spoke English as a second language.	International students also expressed how practice education assisted in their understanding of the Australian hospital system, which was different from their home country.	Increased cultural sensitivity/training for clinical supervisors. The provision of a detailed orientation to the practice placement, including the physical site, available facilities (for example, tea and coffee, bathrooms, and parking), and the service delivery framework.	The sample of international occupational therapy students may not be representative of all International Students from Asian countries.
		Semistructured interview				
Law, et al. (2022). United Kingdom *Scandinavian Journal of Occupational Therapy*	To explore the opportunities and challenges experienced by a student group in practice education. It also aims to examine how the students can be supported to facilitate a high‐quality learning experience.	Phenomenological research design	Six international students enrolled in preregistration OT programs in the United Kingdom	Students identified language limitations and cultural differences created reduced confidence and inclusion. Students reported self‐directed learning demands were different than prior didactic styles, creating exclusion and difficulty with curricula.	Facilitators included the willingness of peers to share cultural expectations. System‐level policies: proactive disability planning, clear accountability, and team‐based competency focus. Need for cultural orientation and university support	This study had a small sample size. Some participants of this study had limitations in their oral proficiency in English and may have struggled to express certain thoughts or opinions during the interviews
		In‐depth, in‐person interviews were conducted with six students.				
Lim, et al. (2016). Australia *Australian Occupational Therapy Journal*	To explore and describe the experiences of international students from Asian backgrounds studying occupational therapy in Australia.	Phenomenological research design	Eight participants were recruited from two occupational therapy programs at the University of Sydney. 2 = Male 6 = Female 3 = Hong Kong 2 = Malaysia 3 = Singapore 7 = Chinese 1 = Muslim Malay	None of the students reported feeling that they had reached an optimal state of competence and mastery, and the occupational identity of international occupational therapy students included being different and separate from domestic students. The findings in the second theme, “fitting into my new role,” indicated that cultural and language issues are major barriers in both adjusting to the university environment and achieving academic success.	Improved integration with domestic students would benefit students by improving both English language skills and understanding of and comfort with Australian culture. It would also benefit domestic students who maintain close interactions with international students to exhibit greater cultural sensitivity, attitudes, and behaviors that foster international awareness, openness, curiosity, and cooperativeness Educators can consider providing structured opportunities in coursework and assessment for international students to relate their learning to practice in their home countries.	This is a small exploratory study, where participants may not be representative of all occupational therapy students from Asian countries in Australia. The sample was primarily composed of Chinese females. The first author was a peer who may have affected the results.
		Data were analyzed using hermeneutic methods.				
Lindsay et al. (2023). Canada *Disability and Rehabilitation,*	To explore the experiences and impact of workplace discrimination and ableism among healthcare providers and trainees with disabilities.	Scoping review	48 studies met the inclusion criteria, representing 13 participants from six countries over 21 years. The sample sizes of the included studies ranged from 2 to 11,859. 20 in the United States 14 in the United Kingdom 8 in Canada 3 in Australia 2 in Ireland 1 in New Zealand Types of disabilities included nonvisible disabilities, dyslexia, learning disabilities, physical disabilities, mental health disabilities, and sensory disabilities.	Barriers included disability discrimination, hostile work environments, retaliation against a person for participating in activities protected by the statute, stereotyping, and bullying. Institutional ableism included inaccessible environments and physical barriers, a lack of support, and an unsupportive work environment. In health professions, these included difficulty disclosing due to fear of stigma, the impact on health and well‐being, and the impact on job and career	Having more knowledge about people with disabilities could help to reduce stereotypes while improving positive attitudes, empathy, and social inclusion. More support at an institutional and senior leadership level is needed to enhance the inclusion of people with disabilities and particularly engage managers and coworkers to be less discriminatory.	Studies were from six countries, which have different customs and policies for the recruitment and treatment of people with disabilities in the workplace. Therefore, it was difficult to make comparisons. Many studies did not specify a distinct disability but were heterogeneous, and the sample sizes were small.
		Systematic searches of seven databases from 2000 to January 2022 were conducted.				
		Five reviewers independently applied the inclusion criteria, extracted the data, and rated the study quality.				
Luong, et al. (2023) United States *Journal of Occupational Therapy Education*	To explore how occupational therapy students′ perceptions of racism and ethnic discrimination impacted their educational experiences.	Mixed method design	Participants included 226 students aged 18–60 years old enrolled in occupational therapy entry‐level, postprofessional, or assistant programs throughout the United States	Barriers included racism and ethnic discrimination in their educational setting. Though students reported having experienced racism or ethnic discrimination within the classroom and fieldwork settings, classroom conversations and content related to topics on diversity, equity, and inclusion (DEI) and antiracism were limited.	Recommendations include the following: (a) acquire additional education in DEI‐related subjects; (b) continue to incorporate more conversations related to topics of DEI, racism, and ethnic discrimination in the classroom and fieldwork settings; (c) provide simulated or real‐life hands‐on opportunities and experiences to work with people of color within the community; (d) teach students how to appropriately behave and respond to racism and ethnic discriminatory situations; (e) create program curriculums that focus on DEI and anti‐racism content; (f) host support groups with diverse people to encourage mentorship between students, practitioners, and community members; and (g) diversify course content to include images, perspectives, and stories of people of color.	The students in the study may not represent the general student population outside of OT or OTA programs; therefore, they cannot be generalized to other student populations. The 20‐min length of the survey may have been a limitation. Also, data were not collected through an interview that would have allowed participants to provide clarification and elaborate meanings of their responses. Not all students were minoritized.
		An anonymous web‐based survey that included a demographic questionnaire, the Brief Perceived Ethnic Discrimination Questionnaire‐Community Version (PEDQ‐CV), and open‐ended survey questions.				
		Mean +/− standard deviation was computed for quantitative variables and frequency for ordinal variables.				
Otuniga & Chichaya (2025). United Kingdom *Irish Journal of Occupational Therapy*	To explore the experiences of Black students enrolled in preregistration occupational therapy programs at a university in the United Kingdom.	Phenomenological research design	Seven Black occupational therapy students were selected using purposive sampling.	Barriers identified (1) horrible peer support from White students, (2) lack of Black representation, (3) difficulty reporting racial issues, (4) being the only Black person on placement, (5) lack of educator professionalism, and (6) hesitancy to report racial issues.	Facilitators included (1) positive Black peer support system and (2) supportive lecturers. Recommendations for educator training, Black educator representation, and a better racial support system.	Conducting interviews via Microsoft Teams may have felt detached for the participants and distorted the conversation′s organic flow, body language, and facial reactions. UK literature is scarce, focused on Black OT students, so literature was drawn from other countries.
		Semistructured interviews were conducted.				
		Thematic analysis resulted in four themes.				
Penman et al. (2021). Australia *Journal of International Students*	To evaluate a transition program created by three disciplines in the health professions addressing international student health and well‐being in Australia.	Commencing students and senior student mentors participated in a four‐session program of activities to reflect on their current study/work practices and learn self‐management strategies.	15 participated in in‐depth interviews. Postgrad dietetics course = 1 Postgrad nursing course = 7 Undergrad nursing course = 2 Undergrad OT course = 5 Mainland China = 8 Hong Kong = 3 Malaysia = 2 Taiwan = 1 Philippines1 Female = 12 Male = 3	International students reported high levels of loneliness and anxiety, saying that they really struggled to fit into university life and also into the Australian way of life more generally. Despite having been accepted by the university into their courses, they expressed strong feelings of self‐doubt in their ability to complete them. They described how their feelings were also barriers to their full participation in university life, both academically and socially.	Programs need to provide an environment that is beneficial in terms of encouraging positive behaviors and participation, reducing academic pressure, and increasing confidence in communication and interactions with students and staff. Another dimension of this theme is the mentee–mentor relationship that emerged, which can contribute to social and peer learning and the creation of a safe environment.	A small number of international students who volunteered to participate limits generalizability. No comparison group.
		Participants developed plans for coping with cultural, language, academic, and social barriers.				
Pride, et al. (2024). Canada *Diaspora, Indigenous, and Minority Education*	To explore the education experiences of health professionals from marginalized groups in Canada.	Qualitative study Thematic analysis was conducted.	Participants include 35 participants from medicine, nursing, and occupational therapy. Nurses = 9 Physicians = 12 OTs = 14 Racialized = 17 Ethnic minority = 12 LGBTQ+ = 11 Working‐class origins = 10 Disabled = 7	For participants, a sense of not fully belonging was conveyed by seeing few people like themselves in their educational programs, particularly in teaching and leadership roles. Participants described how content relating to marginalized groups was rarely included in curricula, and when it was, it included and/or reproduced negative, harmful stereotypes. In addition to isolation, marginalization by classmates and instructors, as well as gaps and stereotypes presented in the curriculum, participants felt excluded in educational contexts when they lacked the social and cultural capital valued in their programs.	Each participant had their own ways of coping with exclusion and marginalization during their education, such as self‐care, substance use, immersion in study, and activism. Two key things that mitigated harm while helping create a sense of belonging and respect were mentoring and community. A clear need was expressed for the program to turn the lens inward, toward changing long‐standing institutional underpinnings and undermining the exclusionary processes that render some learners as “expected” and “normative” whereas others are constructed as misfits.	Only seven of 35 participants were men, disproportionately in medicine. Nuances that are profession‐specific may have been missed. Heterogeneity of the sample is a challenge for theoretical saturation.
Pride, et al. (2025). Canada *Cadernos Brasileiros de Terapia Ocupacional*	To explore the retrospective educational experiences of Indigenous occupational therapists.	Collaborative study with Indigenous occupational therapists, using both Indigenous and Western methods of inquiry. Stage 1 used individual storytelling sessions to hear about participants′ everyday experiences. Stage 2 consisted of an in‐person sharing circle gathering to build relationships and community, and to refine data from Stage 1	Using purposive and snowball sampling. 8 = first nation 5 = Métis 8 = 0–4 years of practice 1 = 5–9 years of practice 4 = 10+ years of practice 4 = Eastern Canada 5 = Central Canada 4 = Western Canada	Imposed isolation resulted in feelings of being “othered” and illuminated who the “expected” OT student is in these spaces. Loneliness, often due to being imposed isolation, deeply impacted these participants′ sense of belonging in their educational programs. Most participants noted a serious lack of support in their respective programs. Affirmative action or “equity” processes have at times resulted in backlash, largely due to myths relating to being underqualified or getting an unfair advantage.	The occupational therapy profession needs to move towards decolonial Indigenization, which requires divesting of colonial privilege and ideologies towards something dynamic and new. Also recommended was a deep consideration of the value of, and need for, multiple perspectives, and drawing on the expertise and experiences of Indigenous students, occupational therapists, researchers, and educators.	Small sample size Most participants had less than 4 years of practice Stories are not necessarily comparable or generalizable.
		Preliminary themes were presented at a sharing circle.				
Ramirez & Kiraly‐Alvarez (2023). United States *Journal of Occupational Therapy Education*	To identify the supports and barriers to inclusion in occupational therapy (OT) education from the experiences of OT students from historically marginalized groups and the perspectives of OT faculty/staff.	Convergent mixed methods design	Using a purposeful, convenience, and snowball sampling technique, 131 students and 35 faculty/staff completed the survey, whereas 20 students and 11 faculty completed interviews.	Lack of social support to navigate the various components of the application process may prevent students from historically marginalized groups from applying to OT school. Limited financial resources to complete the application process and limited financial support from OT programs prevented students from applying to certain programs. Students reported experiencing various degrees of discrimination by both faculty and peers, from microaggressions and culturally insensitive behaviors to overt racism.	Participants identified that opportunities to observe OT practitioners greatly influenced the desire to ultimately choose to pursue OT as a career. Therefore, it is imperative to continue to work on enhancing the awareness and knowledge of the OT profession and creating more opportunities for observing OT practitioners in action. Students who had social supports (e.g., family, friends, and peers) were more likely to overcome barriers and had more opportunities to engage in higher education. Availability of scholarships and clear information about financial aid may support students who experience financial barriers.	Sample size limits external validity. Use of a convenience sample may have resulted in self‐selection bias and response bias. First author identifies as a member of a historically marginalized group.
		Online survey and semistructured interviews.				
		Descriptive statistics to analyze quantitative data from the survey.				
		Phenomenological thematic analysis was used to analyze qualitative responses from surveys and interviews.				
Razek, L. & Zafran, H. (2022). Canada *McGill Journal of Global Health*	To explore admissions processes related to the inclusion of equity groups within McGill University′s Occupational Therapy program and identify recommendations specific to Black applicants.	Critical intersectionality	73 survey respondents White/Caucasian = 46% Black = 3% East or Southeast Asian = 27% Indigenous = 3% Other = 13% Female = 64.38% Male = 35.62% Linguistic minorities = 13.7% Visible minorities = 28.77% LGBTQ+ = 35.62% Disabled = 31.51%	Barriers identified were as follows: Economic capital: Several survey respondents were worried about going into debt because they decided to pursue OT Social capital: Respondents who identified a lack of community support primarily identified as visible minorities (71.4%) Cultural capital: Challenges related to cultural capital included language barriers as well as lower grades in their previous undergraduate degree.	Many suggestions for the admissions were related to financial aid, with some suggesting scholarships for specific marginalized populations and others requesting a clear financial breakdown of costs involved. Survey respondents who did not feel adequately represented by the admissions process believed that other qualities should have been showcased. Many mentioned interpersonal skills, personality, being well‐rounded, advocacy work, and important life challenges.	Only two students identified as Black, highlighting the overall low number of Black OT students and graduates. Questionnaires needed to be bilingual for students whose first language was French. Perspectives of prospective applicants who chose not to apply or failed the application process were not captured.
		Data were collected from a survey of current students and program graduates. Data analysis, applying a critical intersectional lens, was contextualized in dialogue with five				
		key individuals at McGill and a review of the literature.				
Salvant, S., et al. (2021). United States *American Journal of Occupational Therapy*	To discuss the influence of systemic racism on the profession of occupational therapy.	Phenomenological research	Four sessions between June 18 and July 23, 2020, with approximately 200 participants per session.	Participants consistently reported the experience of being “the only” occupational therapy practitioner or student of color in their educational program or among work colleagues. Participants in all groups shared experiences of present‐day discrimination, bias, and racism, ranging from unintentional microaggressions to overt racism. These acts were perpetrated by faculty, fieldwork educators, colleagues and peers, and clients. The term antiracism has become better known since the racial unrest of 2020, and the concept was infused throughout participants′ sharing of experiences, discussion, and recommendations.	Improve recruitment and retention of BIPOC in the profession by promoting pipeline programs to expose BIPOC youth to occupational therapy. Other suggestions included adopting holistic admissions practices in educational institutions and providing financial support for BIPOC students. Also suggested were to promote programs to encourage BIPOC occupational therapy practitioners to act as mentors, and diversify occupational therapy educational materials and presentations. Participants suggested programs: (1) establish bias reporting systems and antidiscrimination policies that are infused with follow‐up and accountability procedures, (2) create ways for BIPOC occupational therapy practitioners and students to share their experiences of racism or trauma, (3) strategize ways of coping, and connect with groups like this National Black Occupational Therapy Caucus, and (4) assess biases and identify antiracist action steps that include measurable goals, objectives, and timelines at the individual and organizational levels of the profession.	Possible selection and response bias. The use of Zoom, which was not accessible to all in 2020. Themes are open to interpretation.
		The AOTA hosted a series of listening sessions, entitled “Be Heard‐We′re Listening,” to provide a platform for BIPOC persons to discuss lived experiences.				
		Common topics, experiences, and recommendations for action were transferred to an Excel spreadsheet and categorized and summarized to determine emerging themes.				
Serrano‐Diaz et al. (2025). United States *American Journal of Occupational Therapy*	To investigate the inclusion and belonging experiences of minoritized occupational therapy practitioners and students internationally.	Scoping review	31 studies across 17 journals highlighted four distinct minoritized groups (some study criteria included multiple identities): culturally and linguistically diverse people (*n* = 16), people with disabilities (*n* = 8), LGBTQ+ individuals (*n* = 8), First Nations people (*n* = 2).	Managing clashes of one′s own cultural beliefs and values with the dominant discourse and ideology of the profession was often required and identified as a barrier. Normative assumptions around ability, sexuality, gender, race, and class negatively affected the minoritized occupational therapy practitioners′, students′, and educators′ sense of belonging Another finding was how some minoritized groups concealed aspects of themselves or their identities because of feelings of not belonging, not being understood, and not being accepted, or because they feared experiencing outright discrimination or different treatment	Factors attributed to enhancing a sense of belonging included the impact of representation, social support from peers, clinical supervisors, faculty, organizational flexibility, and broader inclusion by the profession.	Only articles in English were included. Findings should be treated as preliminary and applied with careful consideration of the unique social, institutional, and population contexts. Many of the findings come from experiences of marginalization within the OT profession in Westernized, industrialized, and settler‐colonial nation‐states. Certain minoritized groups are missing (e.g., transgender, gender‐diverse, intersex, or asexual or aromantic people, members of the Deaf community, specific cultural groups).
		Using NVivo, the authors inductively led the code development, conceptually clustering aspects of the articles and enriching the themes until all included studies had been coded.				
van Rensburg & Kapp (2014) South Africa *South African Journal of Occupational Therapy*	To describe and analyze the learning journey of Zinhle, a first‐generation university student from an impoverished rural village who studied occupational therapy at a relatively elite South African university.	Narrative design	One participant, a first‐generation university student from an impoverished rural village who studied occupational therapy at a relatively elite South African university.	Significant differences between Zinhle′s home environment and the academic culture and practices of the university contributed to the challenges she experienced and demonstrated her “shifts in consciousness, identity and modes of reflexivity related to severe contextual discontinuity.” However, some of the sources of her distress were institutional practices and assumptions	A sense of self was used as a simultaneous explanation for her academic difficulties and a resource that set her apart from others and placed her in a position to be a future agent of change.	Interviews were conducted via Zoom due to safety concerns regarding the global COVID‐19 pandemic. These interviews may have felt impersonal and may have skewed body language, facial expressions, and the natural flow of conversation. Connectivity issues may also have affected these interviews. The methodology is somewhat flawed as the data are self‐reported and filtered through the participant′s point of view. The final limitation includes researcher bias.
		Qualitative longitudinal analysis was used in a single case study to analyze four interviews conducted with Zinhle over her undergraduate years.				
		Data were analyzed inductively by the two researchers. Thematic content analysis was used to analyze the transcribed interviews.				

**Table 2:** Lived experiences.

Author/year, country, journal	Lived experiences
Aldridge et al. (2023). Canada *Journal of Occupational Therapy Education, 7* (4).	Students felt impacted by systemic racism in the academic and clinical settings. They further reported that their sense of belonging was affected by the lack of Black representation in their OT programs.
Benitez et al. (2022). United States *Journal of Occupational Therapy Education*	Participants identified challenges both inside and outside the classroom, including differences in pedagogical methods, English language proficiency, barriers in daily life, and transitioning into the workforce.
Bevan, J. (2014) United Kingdom *Disability & Society*	Participants experienced negative attitudes from clients/patients and colleagues. Also discussed were negative attitudes toward the university application and interview, and inaccessible environments in the educational setting. Experiences with organizational barriers included bureaucratic policies, guidelines, or organizational structures that made it difficult for participants to access information, directly or indirectly.
Ching, E. & Ammon. A. (2020). United States *Diversity & Equality in Health and Care*	Participants expressed feelings of imposter syndrome that impacted their mental health and increased stress and anxiety within the classroom.
Easterbrook, et al. (2015). Canada *Disability & Society*	Students with disabilities had to legitimize their ability to perform the roles of student and future practitioner.
Edelist et al. (2024). Canada *Medical Education*	Students reported that they feared having their competence questioned based on the disclosure of their disability. Students also feared accommodation requests being denied.
Ford, et al. (2021). United States *American Journal of Occupational Therapy*	Participants described feelings of being an outsider within the educational and professional setting. They also described a lack of support from faculty, peers, and clinical instructors.
Gross, E. et al. (2023). Canada *International Journal of Inclusive Education*	Students reported attitudinal barriers when seeking placement accommodations. A requirement to disclose to request accommodations raised fears of having people determine whether students were capable of practicing.
Hendricks, F. & Toth‐Cohen, S. (2018). South Africa *Occupational Therapy International*	An individual′s positive psychological state of development is characterized by (1) confidence, (2) being optimistic about succeeding, (3) perseverance toward goals and redirecting paths toward goals to succeed, and (4) sustaining and bouncing back and even beyond (i.e., resilience) to attain success.
Jarus, T. et al. (2023). Canada *Medical Education*	Participants experience challenges to their sense of legitimacy and belonging as health providers and negatively impacted social relations among colleagues and peers. Student participants experience their peers as viewing their disability and accommodations as an unfair advantage.
Lalor, A. F. et al. (2019). Australia *British Journal of Occupational Therapy*	International students identified that supportive supervisors and a welcoming environment helped to define a successful clinical placement.
	Placements that facilitated open communication helped students′ transition to practice placement.
Law, et al. (2022). United Kingdom *Scandinavian Journal of Occupational Therapy*	Students reported that the ability to learn how to work in multidisciplinary teams led to personal/professional growth
	International students identified negative experiences when faculty assumed that their experiences were similar to local students.
Lim, et al. (2016). Australia *Australian Occupational Therapy Journal*	Participants described three interlinked and ongoing themes that were essential to the experience of being an occupational therapy student in Australia for these students: (1) discovering and engaging with occupational therapy, (2) fitting into my new role, and (3) anticipating my role at home.
Lindsay et al. (2023). Canada *Disability and Rehabilitation,*	Key trends related to student and practitioner experiences of workplace ableism included rates and types of workplace ableism, which occurred at the institutional and individual level, and the impact of workplace ableism.
Luong, et al. (2023) United States *Journal of Occupational Therapy Education*	Students experienced racism or ethnic discrimination in settings, such as external educational opportunities (e.g., school applications), program curriculum, on campus, and in fieldwork. Fieldwork was the most reported setting where students experienced racism or ethnic discrimination
Otuniga & Chichaya (2025). United Kingdom *Irish Journal of Occupational Therapy*	Four themes include lived experiences of bitter‐sweet experiences, harsh realities on placement, need for intentionality when planning the course, and need for creating safe spaces during practice placement.
Penman et al. (2021). Australia *Journal of International Students*	International students report experiencing higher sociocultural and academic stress when settling into a new university compared with their local counterparts.
	Four themes emerged from the text, highlighting the impact of the program on the participants. These were facilitating adjustment, establishing relationships, gaining new skills and knowledge, and transforming beliefs and behavior.
Pride, et al. (2024). Canada *Diaspora, Indigenous, and Minority Education*	Lived experiences included (1) isolation, (2) normative curricula, (3) marginalization by classmates and instructors, (4) exclusionary social and cultural capital, and (5) coping strategies, which represented routine institutional processes in health professions education, particularly access and admissions, formal and informal curricula, and socialization into professional identities
Pride, et al. (2025). Canada *Cadernos Brasileiros de Terapia Ocupacional*	Indigenous occupational therapists experienced imposed isolation, lack of support, exclusion, a devaluing of merit and skill, and “jagged worldviews colliding” in their programs.
Ramirez & Kiraly‐Alvarez (2023). United States *Journal of Occupational Therapy Education*	Themes include the following: (1) Barriers and facilitators exist while exploring OT as a potential career; (2) Pros and cons in the OT admission process for students from historically marginalized groups; (3) Students from historically marginalized groups experience varying degrees of exclusion and a limited sense of belonging; (4) Many OT programs have good intentions to promote diversity and inclusion through various efforts; and (5) Some OT program efforts may be counterproductive, and more actions must be taken to further promote inclusion and address barriers to inclusion.
Razek, L. & Zafran, H. (2022). Canada *McGill Journal of Global Health*	Lived experiences included several barriers to healthcare admissions and education to OT at McGill, which are not limited to the experiences of Black people, but also affect those who identify with other equity‐deserving groups, including LGBTQ+, neurodiverse, visible minorities, and people from lower socioeconomic backgrounds.
Salvant, S., et al. (2021). United States *American Journal of Occupational Therapy*	Lived experiences and suggestions shared by the participants revealed three broad themes related to areas of need: (1) the lack of diversity and representation of BIPOC at all levels of the profession; (2) the experience of racialized trauma, stress, and fatigue; and (3) antiracism.
Serrano‐Diaz et al. (2025). United States *American Journal of Occupational Therapy*	Four themes were identified through lived experiences: (1) negotiating a normative cultural context, (2) feeling a sense of belonging, (3) interacting with clients, and (4) fostering equity and inclusion.
van Rensburg & Kapp (2014) South Africa *South African Journal of Occupational Therapy*	Two main themes were identified from the data. The first theme, her identity and lived experience as a “rural girl,” emerged from categories of home influences and her values and beliefs. The second theme and lived experience of how she acquired access to the discipline was derived from the categories of English as a language challenge, withdrawal from social activities, and her emerging agency and reflexivity.

It should be noted that “under‐represented” student populations represent a diverse population within themselves; therefore, results should be treated as general guidelines. Every attempt will be made to provide explanation of themes relevant to specific populations, but this was not always possible. In addition, the intersectional nature of some “under‐represented” populations made specific recommendations challenging.

### 3.2. Barriers

Barriers experienced in the professional education experience of diverse students included subthemes related to the effects of systemic or organizational context, lack of department or student support, and the students′ feelings or reactions.

#### 3.2.1. Systemic or Organizational Context

Six articles (25%) informed the theme barriers involving system or organizational context. Examples of systematic or organizational context barriers identified by students of color included perceived admission criteria biased toward upper class White students, for example, increased opportunity for leadership or other health care experiences, such as serving as a rehabilitation technician [22], and a lack of financial support [23]. Other systemic or organizational barriers identified by students with disabilities included perceived ableism [24, 25] and inaccessible environments, such as classrooms and labs with equipment that could not be adapted, and fieldwork sites with inaccessible clinical spaces [26, 27].

#### 3.2.2. Department or Peer Student Support

Fifteen studies (60%) focused on barriers identified with regard to department or peer student support. Students from many under‐represented populations discussed having to take on the role of the “diversity person” in class or in the department [28, 29]. Students identified limited diversity, equity, and inclusion (DEI) conversations in the program, a normative curriculum that focused on issues related to middle to upper middle‐class persons [28], language barriers [30–33], and a lack of awareness of marginalized groups [30]. Students of color, students with disabilities, and indigenous students reported feeling isolated [34], experiencing negative attitudes from peers [28, 35], a lack of support from faculty, students, and the educational system [12, 23–25, 28, 35, 36], and vulnerability to authority [26]. Diverse students often felt unsupported by their peers and an increased need to legitimize their ability and competence [23–28, 33, 37].

#### 3.2.3. Student Feelings or Reactions

Twelve studies (50%) discussed how barriers in their educational experience were perceived. Lack of faculty representation of their identity cultures for students of color or students with a disability fostered a student′s sense of not belonging or being an “outsider” [12, 23, 27–29, 31, 35, 38–40]. Students reported that they often felt self‐doubt or experienced imposter syndrome regarding their ability to be successful in the occupational therapy program [30, 41] and experienced racialized trauma [27, 28, 38–40].

### 3.3. Facilitators

#### 3.3.1. Systemic or Organizational Context

Four studies (16%) looking at how systematic or organizational context facilitated positive student experiences included one study that reported how holistic admission practices increased recruitment of diverse students through consideration of lived experiences from under‐represented groups and elimination of in‐person interviews [22]. Students of color and first‐generation students reported both academic and practice success with support from school resources such as access to mental health providers, writing workshops, small classes, and workshops on stress management [33, 42]. Creation of spaces to discuss diversity and inclusion by diverse faculty was also identified as beneficial to students of color [42], as was the importance of having legislation in place for students with a disability to allow diverse students to access the academic and fieldwork settings [27].

#### 3.3.2. Department or Peer Student Support

Five studies (20.8%) described department or peer student support that facilitates positive student experiences. International students and students of color identified that having mentorship opportunities with mentors of the same cultural background and/or lived experience promoted open dialogue, provided a sense of relief and community, and fostered inclusion [28, 39, 43, 44]. Several studies also demonstrated that support from outside the educational organization reduced feelings of isolation, including connections with national and international professional organizations (e.g., Coalition of Occupational Therapy Advocates for Diversity [COTAD] and National Board of Certification for Occupational Therapists [NBCOT]), social media, diverse student support groups, and the broader community [23, 28, 43].

#### 3.3.3. Student Feelings and Reactions

Six studies (25%) described examples of coping strategies that international students and students of color developed to address feelings of marginalization and exclusion, including self‐care practices, building community outside the educational setting, activism, and shifting values to fit in with peers [28, 30, 31]. Those who used activism as a coping strategy often led conversations around diversity, as well as creating chapters of national and international organizations for diverse occupational therapy groups [29]. Students identified that having previous experiences in settings with limited diversity was beneficial to anticipating and adapting to similar situations they may experience as a student [30]. They also reported that sharing their lived experience with clients and clinical instructors enhanced practice and increased their ability to relate to and build rapport with clients during fieldwork placements [26, 27].

### 3.4. Strategies to Address Student Safety and Inclusion

#### 3.4.1. Systemic or Organizational Context

Ten articles (41.7%) included strategies such as the creation of culturally sensitive materials and clear accommodation processes for admission to increase feelings of belonging for students of color and students with disabilities [12, 44]. Suggestions were also made for the development of safe spaces and support services that address racism and other “isms” such as ableism and sexism [38, 42]. Increasing DEI conversations for administration, faculty, and students, including awareness training and listening sessions, was recommended to increase understanding, inclusion, and advocacy for systemic and organizational support of students of color, students with disabilities, and international students [12, 23, 27, 30, 34, 39, 40, 44].

#### 3.4.2. Department or Peer Support

Fifteen articles (60%) identified strategies involving department or peer support to facilitate students′ positive experiences. Accessing support from national and international professional organizations (e.g., COTAD and Black Caucus) that support diversity was recommended to enhance belonging, as well as purposive recruitment of department faculty and students of color or who have a disability [23, 27, 29]. Intentional support of diverse students at orientation [45], creation of support services to address safety and inclusion for under‐represented student populations of color [38], activities that integrate international students [32], and alumni mentorship programs [42, 44] were also identified as strategies to address isolation of students of color and international students [46].

Within the professional education program, reworking of competencies and transparent, competency standards helped address feelings of incompetence and assumptions about competence for students with disabilities and international students by faculty, peers, clinical supervisors, and the students themselves [25, 26, 30]. Reworking of the curriculum to include extended or modified programs was also recommended to increase success in the program for students who may not be able to afford a full‐time program, such as first‐generation college students [33]. Use of critical reflexivity and listening sessions was suggested to increase knowledge of faculty and students about issues faced by students of color [26, 28, 38, 39]. Finally, advocacy by faculty and peers was suggested to address policy inequities experienced by students with disabilities and international students (and ultimately practitioners) [24, 27, 44].

## 4. Discussion

This scoping review describes the complex and intersectional factors that contribute to the lived experience of diverse occupational therapy students, such as students of color, students with disabilities, first‐generation students, and international students during their professional occupational therapy education. Themes developed included the barriers and facilitators to inclusion, as well as the strategies recommended to incorporate facilitators to address the barriers faced by students. Although some barriers and facilitators are relevant to a singular population, most identified through the literature apply to all under‐represented student populations. However, the extent to which these facilitators and strategies are currently used to address identified barriers is unclear. In addition, since a methodological quality assessment was not conducted and typically not required of a scoping review, recommendations should be interpreted cautiously.

### 4.1. Common Facilitators and Barriers to Safety and Inclusion

The included sources demonstrate that the barriers experienced by diverse occupational therapy students are deeply embedded within systemic, organizational, and educational structures. In some cases, as with students of color, these barriers include lack of belonging and increased isolation. With students with disabilities, these barriers may include lack of accommodation. These experiences produced significant emotional labor related to the need to perform “invisible work” to create their own belonging [6], which negatively affected belonging and academic persistence.

Despite these challenges, the review also highlighted several key facilitators that support safety, inclusion, and success. Holistic admissions processes that consider the students′ full backgrounds, clear accommodation pathways, and access to appropriate institutional supports (e.g., mental health services, small group learning, and writing support) were identified as meaningful organizational enablers here and in other studies [47–49]. Students often drew on their own strengths, including self‐advocacy, collective activism, and creating supportive peer communities, to counteract feelings of exclusion. They are also more able to navigate complex cultural contexts in education and practice [31], countering the deficit narrative and problematizing that often accompanies discussion of their place in the profession.

### 4.2. Strategies to Support the Safety and Inclusion of Occupational Therapy Students

Studies explored entry into and experiences within occupational therapy professional programs. While the focus most commonly was on the experiences of students during their professional education, there were several studies exploring the practices leading to the entry of students into these programs, and how these procedures impacted certain groups. Four articles either reported the experiences of students during the entry process or interviewed students about their recommendations for the admissions process [22, 23, 27, 39, 46]. One of the common factors mentioned was the financial requirements for entering occupational therapy. Students were concerned about the costs of the program and required greater information initially about the tuition to make an informed decision before applying [22, 46]. Students also reported concerns regarding the costs associated with applying to professional programs, creating a barrier before they had even applied [27]. In the United States, tuition costs continue to be the greatest barrier to entering occupational therapy programs [50]. While most of the studies reporting on the admissions process were conducted in the United States [23, 39, 46], one study suggests that this may be a more global issue, with greater international research required and action needed to address this inequity where it exists [22]. One of the common recommendations for increasing accessibility to occupational therapy was employing an admissions process that considered more than just academic results [22, 23, 39]. Research and clear recommendations with methodological rigor are needed regarding the most inclusive and fair way of ensuring that greater equity is achieved in the entry process and reducing barriers for diverse students.

While professional bodies in the United Kingdom, Canada, the United States, and Australia indicate the importance of inclusion within the profession, there is clearly a disconnect between intentions and reality [23, 27, 28, 33, 39, 40, 46]. Results of this review suggest that if potential “under‐represented” students believe that they will have a positive experience and can see themselves reflected within the student cohort and faculty, and profession, they may be more likely to apply, leading to greater diversity in educational programs and the profession [28, 39, 46].

There were multiple systems‐level opportunities identified that could improve the experiences of students at an individual level. Some examples included reflecting diversity in curriculum content [27, 31, 45], employing diverse faculty and staff [23, 38], and making content and systems universally accessible to reduce the burden on students with specific needs [24, 27, 36]. Universal design principles have been mentioned as imperative in university education, because if accessibility is addressed for some, it can improve the experience for all [24, 27, 36]. Creating systems‐level change has the potential to improve the educational experience for all students, creating a more inclusive and accessible environment that benefits everyone [24, 27, 36].

The key role played by faculty was also emphasized in the included sources, which advocated for intentional recruitment of diverse academics. Strategies suggested in the literature included recruiting diverse faculty, creating safe spaces for students, and increasing awareness for faculty and peers about issues faced by diverse students [24, 27, 36]. A large study across multiple courses and disciplines [51] highlighted a significant but indirect impact on student success and progression to graduation from alignment between student and faculty racial and ethnic composition. While systems‐level change is required, mentorship was one of the strategies commonly mentioned in the literature. Increasing the DEI capacity of nondiverse faculty is also recommended, and several approaches to its achievement have been explored in the literature [52–54].

### 4.3. Intersectionality and Diverse Occupational Therapy Students

While many studies explored the experiences of students from different identity groups, most included sources did not specifically discuss the experiences of diverse students using an intersectional lens. The concept of intersectionality is emerging as a critical perspective in occupational therapy and has a growing presence in practice, education, and research [55]. Research commonly explored the experiences of students who identified with a specific identity group, for example, students with disabilities [29, 30] or people of color [42]. The research aims of the studies identified were commonly focused on a specific group, and there was little exploration of the impact that intersecting identities may have had on students′ experiences. This has the potential to miss the complexity of people′s experiences that could exist when examining the experiences with these groups. It also limits the ability to analyze findings between different groups, with limited demographic information provided about the samples included. Despite the lack of direct reference to intersectionality in the reviewed articles, the authors provide an original contribution to the literature, by showing that many of the experiences identified in the included sources (e.g., having to “prove” competence and lack of belonging) were consistent across multiple marginalized student groups (e.g., race, ethnicity, ability, and gender). This confirms a core concept within intersectionality by highlighting how the interactions between different identities within the same person can introduce a complexity that may be hidden when groups are considered in isolation [56]. It is clear that the issue of intersectional experiences should be further explored.

## 5. Limitations

Although results from this scoping review were consistent in the identification of facilitators, barriers, and solutions, it is not without limitations. One of the limitations of this review was that sources in languages other than English were not reviewed, as the research team was English‐speaking only and there was insufficient resourcing to locate and translate texts in other languages, limiting the transferability of findings to certain parts of the world. Additionally, care was taken to incorporate terms that reflected as many diverse groups as possible in the search strategy, and the search strategy was reviewed on multiple occasions by a librarian. However, it is possible that certain groups may have been inadvertently missed, which may have impacted the articles that were located and limited the comprehensibility of the search. Within the literature, there were different terms used for “diverse” that added complexity to the search strategy and data analysis. Some common terms used included marginalized, minoritized, under‐represented, and diverse. The use of minoritized and marginalized reflects the systemic power dynamics and the exclusion and disadvantage experienced by certain groups [57, 58]. Alternatively, under‐represented was also commonly used and reflects the small number of people from certain groups but does not reflect the disadvantage experienced by some groups. In addition, some members of the research team did not identify as being a member of any of the diverse groups included in this study, and although the researchers had discussions as a team and worked collaboratively, reviewing each other′s work, this may have influenced the interpretation of the findings. Most articles were not located by using the traditional database keyword search strategy and were instead located using citation searching through Connected Papers that accessed articles not indexed in the traditional databases. This suggests that articles covering DEI topics may not be published in traditional journals, and strategies used to search for articles on these topics should consider this, incorporating additional steps to locate these articles. Publications practices should also incorporate articles on these topics to improve dissemination.

Finally, as mentioned previously, because a methodological quality assessment was not conducted, consistent with scoping reviews, findings and suggestions are considered a starting point for further research.

### 5.1. Implications for Occupational Therapy Education and Policy

Multiple implications for occupational therapy education and policy were identified. These implications have been divided into systemic or organizational, course, and individual levels.

### 5.2. Systemic or Organizational Level Implications

To achieve greater diversity in student cohorts, purposeful recruitment strategies are required, with greater support provided in the application process for those who require it. Greater information about the costs associated with study needs to be provided up front, and more inclusive admissions processes are required, providing students with choice about what they choose to disclose about their identity, with holistic admissions mentioned as one strategy to support greater diversity and inclusivity [22, 23, 27, 39, 46].

Once students have entered the university system, there need to be easily accessible accommodations and support provided for students with disabilities [24, 27, 36]. Staff require training on providing safe and inclusive environments. There need to be educational frameworks developed that accommodate, highlight, and support people from diverse backgrounds [27, 31, 45]. Students also need to be made aware of and encouraged to make connections with professional organizations focused on issues of diversity to support inclusion [23, 29].

### 5.3. Program Level Implications

Strategies are required to reduce isolation and support inclusion. Suggestions to foster inclusion were to facilitate opportunities for mentoring with mentors from similar diverse backgrounds, especially emphasized by students of color. Increased representation within curricula was also suggested [23, 42]. Students suggested that opportunities should be provided to raise the awareness of diverse students′ experiences [27, 38]. It was also suggested that within occupational therapy, competencies need to be changed to address incorrect assumptions about the competence of diverse students, such as students of color, international students, first‐generation students, and students with disabilities [25, 26].

### 5.4. Individual Level Implications

Some approaches that students found useful included finding opportunities to share their lived experiences with peers and mentors [23, 37]. They also suggested employing self‐care strategies [31]. Though individual strategies may support withstanding the system as it is currently, there was emphasis on the importance of creating change at a program and system level [31].

## 6. Conclusion

This scoping review explored how the literature represents the lived experiences of diverse occupational therapy students during their professional education. Recent literature continues to show systemic, organizational, and departmental barriers to student success, resulting in discrimination and reduced feelings of not belonging. Facilitators include mentorship, revised admission criteria, and increased awareness by faculty, peers, and clinical instructors of issues experienced by diverse students. Research is becoming more prevalent but still limited. Future research should examine how interventions at the program level can improve the experiences of diverse students and how to strengthen inclusive student recruitment practices, ultimately improving the experiences of future clinicians and the clients we serve.

## Author Contributions

All authors contributed to the scoping review protocol manuscript. All authors contributed to the search strategy design, data analysis plan, and writing and/or editing the scoping review protocol.

## Funding

No funding was received for this manuscript.

## Disclosure

This paper has been previously published in Research Square [59].

## Conflicts of Interest

The authors declare no conflicts of interest.

## Supporting Information

Additional supporting information can be found online in the Supporting Information section.

## Supporting information


**Supporting Information 1** Supporting Information document II describes the search process followed by the researchers to inform this scoping review.

**Supporting Information 2** Supporting Information document III describes the instrument used to extract the data used for this scoping review.

## Data Availability

The data that support the findings of this study are available in the Supporting Information of this article.
