# Supplementary material for: Lived Experiences of Occupational Therapy Students From Under‐Represented Backgrounds in Professional Educational Programs: A Scoping Review
Source: Occup Ther Int. 2026 Aug 18;2026:8173486. doi: 10.1155/oti/8173486 (PMC13483094; doi:10.1155/oti/8173486)
Supplement: Supplementary file 1 [file OTI-2026-8173486-s001.docx]

**Supplementary Document III**

### Data extraction instrument

| Authors | Include author details |
| --- | --- |
| Title of source | Include the title of the article |
| Publication | Where was this source published? (include the title of the journal). If it’s another type of source, include the organisation and webpage if relevant. |
| Year | Include the year the article was published |
| Date data were collected | Include the time period in which the data were collected. If there is no date published, or this doesn’t apply, write NA. |
| Type of evidence source | Include the type of source e.g. primary research: peer-reviewed research article, evidence syntheses: narrative review, systematic review, scoping review, etc., conference abstract, webpage, report, theses, discussion article, etc. |
| Country | Extract information about where the population investigated is located geographically (city if possible, and country). |
| Aim | Include the aim of the study (as reported in the article). |
| Research methods used | e.g. qualitative – phenomenology, or mixed methods – exploratory sequential. |
| Participants | Include details of the population including any demographic data (this should be relating to the occupational therapists/students), e.g. occupational therapy students in remote Australia. If other populations were also included, record these details here too (e.g. nurses, physiotherapists etc.). |
|  | If multiple populations were included in the sample (e.g. nurses and occupational therapists), can data relating to occupational therapists/students be extracted separately. Yes/no. |
| Sample size | e.g. 6 |
| Concept | Extract details relating to experiences – include themes/deductive framework used. |
|  | Extract details of any facilitators or barriers to safety, inclusion, and/or justice. |
|  | Extract details of any strategies or resources that support occupational therapists/students safety, inclusion and/or justice. |
| Context | Extract information about the under-represented population/s. Include age, sex, gender, class, identifying with/without disability, neurodiversity, cultural group, sexual orientation, religion, socio-economic status, remote/rural. Note any intersecting identities. |

Data extraction instrument adapted from: Pollock, D., Peters, M.D.J., Khalil, H., McInerney, P., Alexander, L., Tricco, A.C., Evans, C., de Moraes, E.B., Godfrey, C.M., Pieper, D., Saran, A., Stern, C. & Munn, Z. (2023). Recommendations for the extraction, analysis and presentation of results in scoping reviews. *JBI Evidence Synthesis, 21*(3), 520-532. https://doi:10.11124/JBIES-22-00123
