# Supplementary material for: Lived Experiences of Occupational Therapy Students From Under‐Represented Backgrounds in Professional Educational Programs: A Scoping Review
Source: Occup Ther Int. 2026 Aug 18;2026:8173486. doi: 10.1155/oti/8173486 (PMC13483094; doi:10.1155/oti/8173486)
Supplement: Supplementary file 2 [file OTI-2026-8173486-s002.docx]

### **Supplementary Materials II: Search strategy**

The following search strategy was used in Medline (via EBSCO Host). The search terms and Boolean operators are detailed below. The limits will include English, and 1^st^ January 2014 to 2024. The search was conducted on 30^th^ July 2024.

| **Search terms** | **Results** |
| --- | --- |
| MH “Native Hawaiian or Other Pacific Islander” or MH “oceanians+” or MH”cultural diversity+” or MH “workforce diversity” OR AB (("cultural* divers*" or "ethnic* divers*" or "racial* divers*" or ((cultural* or linguist*) N5 divers*) or underrepresent* or "international student*" or "international* educat*" or (student* N5 disabilit*) or "disabled student*" or "gender equity" or "gender discriminat*" or "gender bias" or “gender divers*” or LGBTIQ+ or LGBTQI+ or LGBTQ or LGBT or lesbian* or gay or homosexual* or bisexual* or transgender or queer or migrant* or refugee* or "language* other than English" or rural or remote or isolated or country or Indigenous or "Torres Strait Islander" or "First Nation*" or Aborigin* or "mature age" or disadvantage* or “first in family” or "low ses" or "low socio-economic status" or poverty or Maori or Pasifika or black or colour or color)) OR TI (("cultural* divers*" or "ethnic* divers*" or "racial* divers*" or ((cultural* or linguist*) N5 divers*) or underrepresent* or "international student*" or "international* educat*" or (student* N5 disabilit*) or "disabled student*" or "gender equity" or "gender discriminat*" or "gender bias" or “gender divers*” or LGBTIQ+ or LGBTQI+ or LGBTQ or LGBT or lesbian* or gay or homosexual* or bisexual* or transgender or queer or migrant* or refugee* or "language* other than English" or rural or remote or isolated or country or Indigenous or "Torres Strait Islander" or "First Nation*" or Aborigin* or "mature age" or disadvantage* or “first in family” or "low ses" or "low socio-economic status" or poverty or Maori or Pasifika or black or colour or color)) | 2,734,976 |
| MH “occupational therapy” or TX (“occupational therap* profession*” or “occupational therap* student*” or “occupational therap*”) | 50,051 |
| MH “Workforce+” or MH “Health workforce” or MH or TX ((transition* or “leav* N2 school” or “career chang*” or recruit* or “new graduate*” or novice) NOT intervention*) | 1,106,341 |
